# Barriers and enablers to obesity prevention in female-only high schools in Riyadh: a qualitative study exploring healthy eating, physical activity and school-based interventions using the COM-B model

**DOI:** 10.1186/s12889-026-26568-1

**Published:** 2026-02-20

**Authors:** Sarah Aldukair, Jayne V. Woodside, Khalid Almutairi, Laura McGowan

**Affiliations:** 1 Centre for Public Health, School of Medicine, Dentistry and Biomedical Sciences, Queen’s University Belfast, Belfast, UK; 2 Department of Health Sciences, College of Health and Rehabilitation Sciences, Princess Nourah Bint Abdulrahman University, Riyadh, Saudi Arabia; 3 Department of Community Health, College of Applied Medical Sciences, King Saud University, Riyadh, Saudi Arabia

**Keywords:** Obesity prevention, Female adolescents, Qualitative study, Healthy eating, Physical activity, COM-B model, Behaviour change, School-based interventions, Saudi arabia

## Abstract

**Background:**

In the Kingdom of Saudi Arabia (KSA), adolescent health is suboptimal. Findings reported that 79% of youths aged 15–29 were physically inactive with 30% living with overweight or obesity. Poor dietary habits further complicate the obesity epidemic. Schools are promoted as key settings for obesity prevention, yet little is known about female-only high schools. This study explored barriers and enablers to healthy eating (HE), physical activity (PA), and obesity prevention school-based interventions (SBIs) through conducting focus group discussions (FGDs) with students and staff.

**Methods:**

Nine FGDs were conducted across three female public high schools in Riyadh from varying deprivation levels; six with 37 students (aged 16–17) and three with 19 staff members. A semi-structured topic guide, informed by the COM-B model, explored capabilities, opportunities, and motivations related to obesity prevention. Framework analysis identified key barriers and enablers to HE, PA, and SBIs implementation.

**Results:**

Barriers emerged across all COM-B constructs. Capability-related barriers included lack of trained staff. Opportunity-related barriers were most prominent, including hot weather, curriculum limitations, and built school environment. Staff and students collectively agreed that low student motivation was a key barrier. School staff highlighted structural enablers such as the physical education curriculum, while students identified individual-level motivators including willpower, improved mood, health, and body image. No mutual enablers were identified across staff and student groups.

**Conclusion:**

Female-only high schools in KSA face major barriers to obesity prevention SBIs, with low student motivation emerging as a dominant barrier across staff and student groups. Addressing these barriers through context-specific, multi-level approaches integrating staff and student perspectives is critical for effective SBIs.

**Supplementary Information:**

The online version contains supplementary material available at 10.1186/s12889-026-26568-1.

## Key messages

What is already known on this topic:

- Childhood obesity rates in KSA are among the highest globally and driven by poor dietary habits, physical inactivity, socio-cultural and environmental factors.
- Schools are considered a key setting for health promotion.
- Obesity prevention initiatives within the context of female-only schools in KSA remains underexplored with most evidence originating from Western or mixed-gender settings.
- Female-only schools in KSA operate within unique cultural and environmental conditions (such as gender segregation and limited physical activity opportunities for females).
- This creates a need to further explore how to effectively design and implement SBIs in this unique context.

What this study adds:

- This is the first qualitative study to investigate barriers and enablers to healthy eating, physical activity and school-based obesity prevention interventions implementation among both female high school students and staff in Riyadh.
- By employing the COM-B model, the socio-ecological model and using framework analysis, the study offers a structured and behaviourally informed perspective.
- This study identifies structural and motivational barriers, such as the built school environment, lack of trained staff, curriculum limitations, and lack of student motivation for healthy eating and physical activity.
- It also identifies a range of behaviourally-informed enablers across COM (capability, opportunity and motivation) such as staff cooperation, peer support, the introduction of a physical education curriculum, body image, and health-related motivators.
- These findings provide context-specific understanding of obesity prevention SBIs in Saudi schools.

How this study might affect research, practice, or policy:

- This study highlights the need for culturally appropriate and context-specific obesity prevention SBIs, that address the individual, interpersonal, institutional, community, and public policy levels.
- It supports staff training, improvement of the built school environment, and practical PA in schools.
- The study informs future SBIs research in KSA and Gulf Cooperation Council countries with similar contexts.

**Supplementary Information:**

The online version contains supplementary material available at 10.1186/s12889-026-26568-1.

## Background

Childhood obesity is a major public health challenge globally and particularly in KSA, reporting some of the highest rates of overweight and obesity [1]. The 2019 KSA World Health Survey indicates that adolescent health is suboptimal, reporting 30% of 15–29-year-olds are living with overweight or obesity, with pre-diabetes and raised cholesterol affecting 10% and 39% of respondents, respectively, alongside 79% having insufficient physical activity (PA) [2]. Systematic reviews reveal that sedentary lifestyles and poor dietary behaviours are widespread among adolescents, including high fast-food consumption, low fruit and vegetable intake, breakfast skipping [3–5] and a 2023 scoping review illustrated that approximately 80–90% of adolescents fail to meet recommended daily PA targets [6].

The World Health Organisation (WHO) identifies schools as key settings for promoting healthy behaviours [7, 8]. A qualitative systematic review of 18 school-based studies concluded that schools play a crucial role in obesity prevention by providing opportunities to improve PA and healthy eating (HE) behaviours, however, interventions are often limited in reach or efficacy [9]. In KSA, little is known about the barriers and enablers to effective implementation of obesity prevention SBIs in female schools, where gender-segregation, cultural restrictions, and limited physical education (PE) provision until 2017 represent unique challenges [10–12]. Female adolescents in KSA face additional barriers, including biological predispositions to adiposity [13] and social pressures related to body image [14, 15]. Although KSA’s Vision 2030’s recent policies aim to improve PA opportunities for females, cultural and environmental constraints remain [16].

Limited evidence about barriers and enablers to SBIs, HE, and PA from KSA exists, with most research being conducted in Western contexts [17–20]. Some studies offer insights from Jeddah [21–23], but little is known about female high schools in Riyadh, particularly from students’ and school staff perspectives. Behaviour change models can offer valuable frameworks to conceptualise barriers and enablers to behaviour change, such as the COM-B model, which is derived from a synthesis of 19 behaviour change frameworks and conceptualises behaviour as the interaction of capability (physical skills and psychological knowledge), opportunity (physical resources and social support), and motivation (automatic habits and reflective intentions) [24, 25].

It can be used to guide context-specific intervention design, for example, the Healthy Buddies program employed this model to improve healthy behaviours in elementary schools [26]. Additionally, given the complex myriad of factors which contribute to the development of obesity, the Social Ecological Model (SEM) was employed to provide a comprehensive perspective on obesity risk factors by recognising the multiple layers of influence; individual, interpersonal, institutional, community, and public policy [27]. Focusing on motivation as a key behavioural construct is theoretically informed by the COM-B model, which identifies motivation as a core component of behaviour change, alongside capability and opportunity. Similarly, the SEM emphasises motivation as a key individual-level factor which interacts with wider environmental and social factors to shape adolescent behaviours.

This study explores barriers and enablers to HE and PA from students, alongside exploring barriers and enablers to implement obesity prevention SBIs from staff, guided by COM-B and the SEM.

## Methods

### Design of qualitative FGDs

This qualitative study was comprised of semi-structured FGDs with separate groups for school staff (school staff and principals) and high school students to explore barriers and enablers of HE, PA, and implementing obesity prevention SBIs across high schools situated in areas of varying economic deprivation. Students were drawn from grades 10–11, typically aged 16–17 years.

FGDs were selected to facilitate collective sharing of the lived experience among both participant groups, and given the age of students’ participants, to support peer interaction and engagement [28].

A semi-structured topic guide was developed informed by the COM-B model [24], exploring capabilities, opportunities, and motivations of school staff regarding barriers and enablers of implementing obesity prevention SBIs; and for students regarding barriers and enablers to HE and PA. This qualitative study employed a primarily theory-driven deductive approach, informed by the COM-B model to structure the topic guides to produce as many actionable strategies as possible. FGDs were conducted separately for staff and students; topic guides are provided in supplementary material (i). This qualitative study was conducted and reported in accordance with the Consolidated Criteria for Reporting Qualitative Research checklist (see supplementary material ii) [29].

### Personal and public involvement and engagement (PPIE)

Ensuring research is relevant and framed sensitively is important, especially when working with adolescents on the topic of HE and PA. Given the involvement of high school students in the present study, PPIE was incorporated to obtain adolescent input on the research design, framing and to assess their understanding of the FGD topic guide [30]. Using the researcher’s local networks, a snowballing approach was taken to engage PPIE representatives. Two young people from the target age group (females) joined an informal video call with the researcher [SA] to provide feedback on the topic guide and framing of the research, and revisions were made accordingly. This ensured the study addressed student priorities and aligned with their language/terminology.

### Setting, population, and sampling technique

There are approximately 279 female governmental high schools in Riyadh. Schools were categorised into three deprivation areas; low deprivation schools (LDS), medium deprivation schools (MDS), and high deprivation schools (HDS). Deprivation status was determined based on the price per square metre of land, according to documents from the Saudi Real Estate General Authority [31].

Although the land price per square metre is an indirect measure of deprivation, the research team deemed this proxy measure acceptable given the lack of standardised measures of deprivation in KSA [32].

Schools within each category were randomised in an Excel sheet and numbered, and three schools were drawn randomly from each list using random number generators. The first schools contacted by e-mail from the low and medium deprivation status cohorts agreed to participate. After the first HDS declined participation in the study, a second school was randomly selected from the remaining list and agreed to participate. The FGDs were then conducted in these three female public high schools in Riyadh with differing levels of economic deprivation. Six separate FGDs were conducted with students (two per school) and three with staff (one per school). The target sample size for each FGD was 6–8 participants. Given the age of participants, the nature of the topic and the complexity of the issue being discussed, a smaller group was considered more desirable [33]. Eligible participants included female students aged 16–17 years and school staff at the selected schools who were available during the scheduled FGDs.

### FGDs participants and recruitment

Eligible students were provided with a participant information sheet (PIS) and parental consent forms; participants were randomly selected alphabetically by surname. Staff received a PIS and were invited to participate based on availability and consent. No questionnaires were distributed to participants prior to the study to assess demographic factors. However, a brief description of the roles of school staff is outlined in Table 1.

**Table 1:** School staff roles

School staff roles			
	LDS	MDS	HDS
Teachers	n=5	n=6	n=5
Principals	n=1	n=1	n=1

*LDS*: Low deprivation school, *MDS*: Medium deprivation school, *HDS*: High deprivation school

Each FGD lasted approximately one hour, conducted in Arabic by the lead researcher [SA]. Recordings were transcribed verbatim, translated into English, and back-translated for accuracy by [SA] and verified by a certified translator.

### Data collection and storage

All FGDs were moderated by a female PhD researcher from KSA with training in qualitative methods, and no prior relationship with study participants. To manage group dynamics, ground rules were established at the beginning of the FGDs to ensure equal and respectful participation. FGDs were audio-recorded anonymously using a password-protected device, with recordings saved in encrypted folders labeled by school, deprivation level, and participant type. The master list was kept separately to ensure anonymity.

### Qualitative analysis

The lead researcher [SA] initially read the transcripts repeatedly, and generated codes. Double-coding and discussions with LM and the supervisory team ensured triangulation and consensus on the coding approach. Data were analysed according to COM-B constructs: physical and psychological capability, physical and social opportunity, and automatic and reflective motivation. Codes were categorised as barriers or enablers. During data analysis, all coding was successfully applied and categorised under COM-B model constructs. Codes that overlapped across constructs were identified and resolved during the process of double-coding and triangulation (See supplementary material iii).

A framework analysis was employed to guide the qualitative analysis of FGDs [34]. The primary aim of framework analysis is to recognise, describe, and identify prominent trends present in the qualitative data under investigation and organise them according to the selected model, COM-B, supported by NVivo 12 software.

### Reflexivity

The research team’s expertise shaped study design and interpretation. Dr. Laura McGowan has training in psychology, health psychology, obesity and health behaviour change; Prof. Jayne Woodside in nutrition and public health; Prof. Khalid Almutairi in statistics and public health, and Sarah Aldukair in public health and health education.

FGDs were conducted and moderated in Arabic by a Saudi female PhD researcher (Sarah Aldukair). The moderator’s insider cultural position facilitated access to schools and helped building rapport with female participants.

### Ethical consideration

The study was approved by Princess Nourah bint Abdulrahman University institutional review board and Queen’s University Belfast’s Faculty of Medicine, Health and Life Sciences REC (MHLS 23_62). The Saudi Ministry of Education granted permission to access schools. The ethical approval conformed to the principles embodied in the Declaration of Helsinki. Due to potential sensitivity of HE, PA, and weight-related discussions, participants were provided with a brief leaflet signposting students to information about support services at the end of each FGD. Participation was voluntary, with written informed consent obtained from staff, students, and parents prior to participation. Additionally, it was made clear in the PIS that if participants have chosen to take part in the study, they can change their minds at any time and withdraw from the study without giving a reason.

## Results

Three high schools were recruited to the FGD study and are outlined in Table 2. 

**Table 2:** Number of participants recruited for focus group discussions

School area	Number of FGDs with students	Number of FGDs with school staff	Number of FGDs participants achieved
1 LDS	2	1	Students (*n* = 13 across two FGDs) School staff (*n* = 6)
1 MDS	2	1	Students (*n* = 12 across two FGDs) School staff (*n* = 7)
1 HDS	2	1	Students (*n* = 12 across two FGDs) School staff (*n* = 6)
Total across 3 different schools from 3 different areas of deprivation	9 FGDs		37 students, 19 school staff members

*FGDs*: Focus group discussions, *LDS*: Low deprivation school, *MDS*: Medium deprivation school, *HDS*: High deprivation school

A total of 37 female students and 19 staff members (including teachers and principals) participated across the nine FGDs. Findings are presented using the COM-B framework, highlighting barriers and enablers to HE, PA, and the implementation of obesity prevention SBIs as highlighted in Tables 3 and 4.

**Table 3:** Codes across school staff focus group discussions

Codes across school staff focus group discussions			
Barriers			
School area	COM-B Constructs		
	Capability	Opportunity	Motivation
LDS	-Lack of trained staff and unpreparedness	-Built school environment -Time constraints -Hot weather -Curriculum limitations -Inconsistent initiatives	Lack of students’ motivation for healthy eating and physical activity
MDS			
HDS			

Enablers			
School area	COM-B Constructs		
	Capability	Opportunity	Motivation
LDS	-Perceived school-based intervention feasibility	-Newly introduced physical education curriculum -Presence of a canteen policy	﻿-Importance of obesity prevention -Cooperation of school staff -Presence of staff motivation
MDS			
HDS			

*COM-B*: Capability-Opportunity-motivation=Behaviour, *LDS*: Low deprivation school, *MDS*: Medium deprivation school, *HDS*: High deprivation school

**Table 4:** Codes across students’ focus group discussions

Consistent codes across students' focus group discussions			
Barriers			
School area	COM-B Construct		
	Capability	Opportunity	Motivation
LDS	-Difficulty of healthy eating	-Affordability -Cultural restrictions -Hot weather -Social food habits -Home environment -Transportation -Curriculum limitations -Built school environment	-Lack of motivation for healthy eating and physical activity
MDS			
HDS			

Enablers			
School area	COM-B Construct		
	Capability	Opportunity	Motivation
LDS	-Healthy eating solutions -Physical activity is simple -Presence of willpower	-Presence of walking facilities	-Body shape -Mood and health improvement -Peer-to-peer support -Well-designed school-based interventions
MDS			
HDS			

*COM-B*: Capability-Opportunity-motivation=Behaviour,* LDS*: Low deprivation school, *MDS*: Medium deprivation school, *HDS: *High deprivation school

### School staff perspectives

#### Barriers and enablers relating to capability

In the school staff FGDs, factors relating to capability were consistent across the different schools sampled, with lack of trained staff noted as a prominent barrier.

‘’I have no experience in the field of school-based interventions... I am supposed to be familiar with the subject by taking a short course or training before the beginning of the intervention.’’ (Teacher 2-HDS)

There was less consistency regarding enablers, although each school identified a different enabler; perceived SBIs feasibility in the LDS, adolescent maturity in the MDS, and physical strength in the HDS.

“It is simple; provide fruits, vegetables, and healthy options in the canteen. I don’t think it will be difficult at all...” (Teacher 1-LDS)

“High school students are older than middle or primary school students, so they would be more convinced in obesity interventions because they are always concerned with their body shape, and this can motivate them to be healthier because they want to look beautiful.” (Teacher 2- MDS)

“...students at this age are young and have the physical strength to be active and practice physical activity, but they need a suitable environment for that.” (Principal-HDS)

#### Barriers and enablers relating to opportunity

Factors relating to opportunity were consistent across different schools; with the built school environment as a prominent barrier. The majority of school staff discussed the barriers to PA and HE related to the opportunity of physical environment:

‘’The school environment is not ready at all, at all, at all! Not even the slightest readiness... How can we implement a school program in our school? The environment is not supportive of health programs... because canteens are very unhealthy and there is no gym... the situation of our canteen can hinder positive change, no matter how good the intervention is.’’ (Teacher 6-MDS)

‘’Let’s say the school makes efforts to support healthy choices, but when the student looks at the school environment that contradicts what has been taught, school efforts are ruined. The students may be aware of the importance of healthy eating and physical activity, but the school environment does not support their ideas.’’ (Teacher 4-HDS)

The newly introduced physical education (PE) curriculum emerged as a consistent enabler across different schools:

“The newly introduced physical education curriculum is an excellent addition for the students’’- (Teacher 1-LDS)

“The PE curriculum is a nice addition... and hopefully soon it will include exercising sessions.” (Teacher 3-MDS)

#### Barriers and enablers relating to motivation

Factors relating to motivation were also consistent across schools, with the importance of obesity prevention identified as an enabler:

“I believe that healthy eating and physical activity are very important, especially among teenagers, so believing in the importance of obesity prevention motivates me...” (Teacher 1- HDS)

‘’ The importance of obesity prevention, healthy eating and physical activity, especially for adolescent girls... I strongly believe that maintaining a healthy lifestyle is one of the most important things.” (Principal-LDS)

Lack of student motivation, however, was highlighted as a barrier:

‘’ I would like to add that it is something that entirely depends on the student's beliefs and motivation...students are not motivated to eat healthy or exercise’’ (Teacher 3-HDS)

‘’ I try to motivate my students, but the students are not cooperative with me, and I think the biggest reason is being shy to perform physical activity.’’ (Teacher 4-LDS)

### Students’ perspectives

#### Barriers and enablers relating to capability

In the student FGDs, factors relating to capability were consistent across schools, with difficulty of HE due to taste preferences as the main barrier:

‘’It is very difficult to eat healthy because I have to cut the sugars and refrain from foods I like. Healthy food is not tasty so it will be hard for me to eat it.’’ (Student 1-MDS)

‘’For me following a healthy diet is extremely difficult because I have noticed that my mood is connected to food immensely... For example, if there is salad and burger what would you choose? For sure all of us will choose the burger, it tastes better!’’ (Student 4-LDS)

Enablers were consistent across schools and included awareness of HE solutions, recognition that PA can be simple, and the presence of ‘willpower’ or motivation was considered important from their perspective:

‘’If eating healthy it is a gradual diet and there is no sudden abstinence from unhealthy foods, it will become less difficult.’’ (Student 1-MDS)

‘’I don’t consider physical activity difficult; it can be for 15 minutes a day only... the most important thing is to devote time to it.’’ (Student 2-HDS)

‘’If a person has the determination and willpower, physical activity will become easy... and gradually it will become doable’’ (Student 1-LDS)

#### Barriers and enablers relating to opportunity

Factors relating to opportunity were also consistent across schools; barriers included affordability, cultural restrictions, hot weather, social food habits, unsupportive home environments, transportation challenges, curriculum limitations, and inadequate built school environments:

‘’Healthy food is expensive and unhealthy food is available and cheap... Healthy alternatives are always expensive.’’ (Student 5-LDS)

‘’It is socially unacceptable for a girl to go out to walk in the neighborhood’’ (Student 5-HDS)

‘’ I like walking and I don't think it is hard but it needs a nice weather and the weather in our country is hot almost all year long.’’ (Student 6-MDS)

‘’In social gatherings it is hard to refuse the food served because it is considered impolite...There is a lot of pressure to eat at social events and the food is extremely unhealthy’’ (Student 4- LDS)

‘’At home, vegetables and fruits aren't always available. Healthy food isn't available.’’ (Student 2-LDS)

‘’Walkways are nearby, but we can't just walk, we must drive to that place... which is an obstacle, transportation is not always available.’’ (Student 3-LDS)

‘’ Although we study physical education but the problem is we don't apply it. We only study it theoretically.’’ (Student 1-MDS)

“The canteen is extremely unhealthy, and we don't do any physical activity.’’ (Student 1-LDS)

The presence of walking facilities was identified as a consistent enabler across different schools:

‘’I like walking and I don't think it is hard, but it needs a nice weather and the weather in our country is hot almost all year long. However, there are alternatives like the air-conditioned malls.’’ (Student 3-MDS)

#### Barriers and enablers relating to motivation

Factors relating to motivation were consistent across schools, with the lack of motivation to engage in HE and PA as a barrier.

‘’ I just don't have the motivation to prevent myself from eating unhealthy food.’’ (Student 1- HDS)

‘’ I have no motivation to eat healthy or exercise’’ (Student 3-LDS)

Consistent enablers included the importance of body shape, mood and health improvement, peer-to-peer support, and well-designed SBIs that include competitions.

‘’ When my body shape becomes nice, I feel that I have succeeded.’’ (Student 6-MDS)

‘’When I eat healthy food and exercise that would elevate my physical and mental health. Also, when that reflects on my skin, my hair and my looks.’’ (Student 1-HDS)

‘’If we all eat healthy food together as friends, we will certainly get excited.’’ (Student 1-LDS)

‘’There should also be competitions between schools to boost competitiveness between students’’ (Student 6-MDS)

### Mutual findings

Across both groups, mutual barriers were consistently identified (Table 5); extreme heat, curriculum limitations, built school environment, and the lack of students’ motivation for HE and PA. No mutual enablers were identified across both groups.

**Table 5:** Mutual codes between students’ and school staff focus group discussions

Mutual codes between students’ and school staff focus group discussions			
Barriers			
School area	COM-B Construct		
	Capability	Opportunity	Motivation
LDS	-None	-Hot weather -Curriculum limitations -Built school environment	- Lack of motivation for healthy eating and physical activity
MDS			
HDS			

Enablers			
School area	COM-B Construct		
	Capability	Opportunity	Motivation
LDS	-None	-None	-None
MDS			
HDS			

*COM-B*: Capability-Opportunity-motivation=Behaviour, *LDS*: Low deprivation school, *MDS*: Medium deprivation school, *HDS: *High deprivation school

### Summary of barriers and enablers mapped to the SEM

**Fig. 1:**
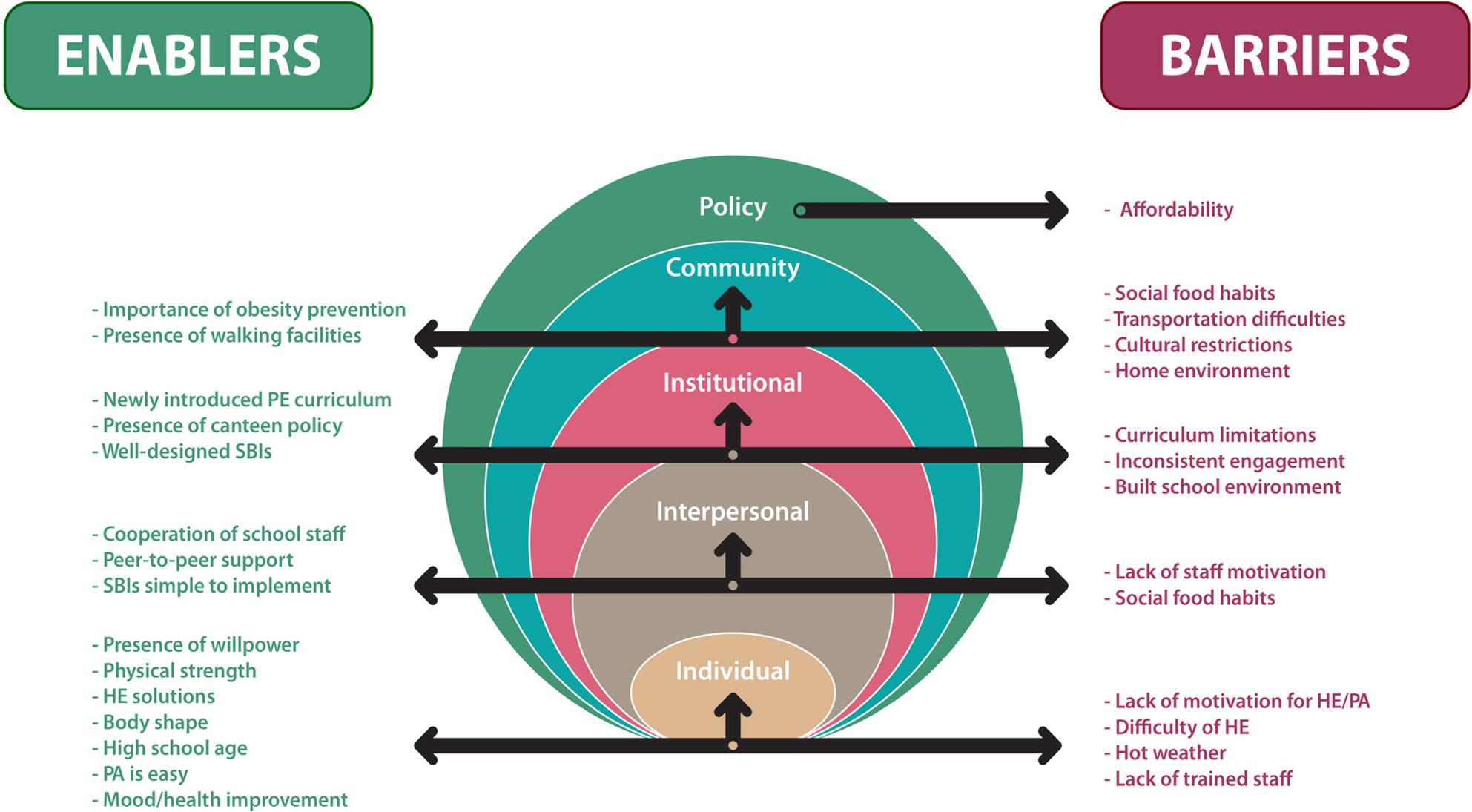
Summary of barriers and enablers mapped across the Social Ecological Model [27]

COM-related barriers and enablers emerged at multiple levels of the SEM: individual (lack of motivation, difficulty of HE, hot weather, lack of trained staff, presence of willpower, physical strength, HE solutions, body shape, adolescent maturity, PA is easy and mood and health improvement), interpersonal (lack of staff motivation, social food habits, cooperation of school staff, peer support and SBIs are simple to implement), institutional (curriculum limitations, inconsistent initiatives, built school environment, newly introduced PE curriculum, presence of canteen policy and well-designed SBIs), community (social food habits, transportation, home environment, cultural restrictions, walking facilities and importance of obesity prevention), and policy (affordability). This multi-level perspective highlights the importance of addressing obesity influences beyond the individual-level to achieve behavioural change, as illustrated in Fig. 1.

## Discussion

This study identified key capability, opportunity and motivation-related barriers and enablers to implementing obesity prevention SBIs targeting HE and PA among female students and staff in Riyadh, KSA. In school staff FGDs, key barriers included lack of trained staff (capability), curriculum limitations, built school environment, time constraints, hot weather, inconsistent initiatives (opportunity), and low student motivation for HE and PA (motivation). These barriers were consistent across schools of differing deprivation levels. Enablers included student age and strength, perceived SBIs feasibility, newly introduced physical education curriculum, canteen policy, staff cooperation and motivation.

In student FGDs, barriers to HE and PA included difficulty and affordability of HE, cultural and environmental factors, transportation, and lack of motivation. Enablers included practical HE solutions, ease of PA, presence of willpower, presence of walking facilities, body image, mood and health improvement, peer support, and well-designed SBIs. Both barriers and enablers were consistent across schools from varying deprivation levels. Both groups identified mutual barriers: hot weather, curriculum limitations, poor school infrastructure, and low student motivation. No mutual enablers were found.

The key findings align with previous research emphasising the environmental, structural and motivational barriers to HE and PA in schools [17–20], while also emphasising context-specific issues relating to Saudi female schools, such as gender-segregated environments, unsupportive school infrastructures, the availability of PA, and policies unique to female students [15]. This study adds to the literature by highlighting the perspectives of both students and staff, reinforcing the need for tailored, multi-level interventions.

Despite evidence on the effectiveness of SBIs to prevent obesity [7–9], a significant number of schools fail to successfully implement effective interventions due to several barriers [35]. This study highlights barriers and enablers in Saudi female high schools, underscoring the need for context-specific approaches to strengthen schools’ role in obesity prevention, which go beyond targeting education alone and focus on multiple capability, opportunity and motivational strategies to improve HE and PA behaviours.

School staff identified the lack of training, heavy workloads, and time constraints as barriers to the implementation of obesity prevention SBIs. This aligns with previous findings identifying time constraints as a major barrier to implementing effective SBIs [36]. School staff and students jointly agreed that the newly introduced physical education curriculum was frustrating as it only contained theoretical information, stressing the need for an applied component to make it more useful. This is consistent with findings from a systematic review revealing that implementation of diverse PA activities enhances intervention impact [37]. The built school environment was criticised for lacking adequate PA facilities and HE offerings, which aligns with two Saudi studies revealing organisational challenges in creating school environments conducive to PA and HE [21, 22]. Extreme weather was also a major barrier to PA, given the open structure of Saudi school buildings and the dangers of exercising outdoors in the heat.

Indeed, previous research conducted in Brazil has shown that poor weather was one of the main perceived barriers to PA among adolescents [38]. Finally, both school staff and students highlighted low student motivation as a persistent barrier, compounded by the lack of sustained health initiatives, often limited to one-time events.

Despite the presence of several barriers, school staff identified enablers. Enablers varied by deprivation level; In the LDS, staff perceived SBIs as feasible to implement, though systemic barriers remained. In the MDS, adolescent maturity was seen as an enabler, since students are more health and appearance conscious. In the HDS, staff highlighted students’ physical strength, provided the environment supports PA. Across all schools, staff valued the introduction of the PE curriculum, the canteen policy, the importance of obesity prevention, and their own cooperation and motivation as enablers to support future interventions.

Students consistently identified the difficulty of HE as a major barrier, linking it to taste preferences, affordability, and unsupportive home environments. Cultural restrictions were reported as barriers to PA, particularly the limitation on females walking alone in neighborhoods. Previous research explicitly identified cultural restrictions as barriers to PA among Saudi females [12]. Students also identified social gatherings as barriers to healthy eating, consistent with previous research revealing that such settings often hinder HE among young people [39]. Transportation challenges limited access to PA facilities, reflecting Riyadh’s car-dependent infrastructure. Extreme heat further discouraged outdoor PA. These barriers highlight the influence of cultural, environmental, and structural factors on adolescent health behaviours.

Conversely, students identified several enablers to healthier behaviours. They highlighted dietary solutions such as moderation, gradual diet changes, and provision of healthy options in schools as enablers to HE. This supports earlier findings that offering healthy food in school canteens enables HE [21]. While HE was perceived as difficult, PA was seen as relatively easy if time, determination and willpower were present. The presence of walking facilities, such as in neighborhood walkways and in malls, were considered supportive environments enabling PA. Students also highlighted body image, mood, and health improvement as key motivators to HE and PA, aligning with previous findings that young adults are motivated by appearance, wellbeing, and health-related factors when making dietary choices [40]. Similarly, findings from a qualitative study revealed that motivators for PA included both body image and health improvement among Saudi females [21]. Peer support was emphasised as a powerful enabler, with students noting that engaging in HE and PA with friends was more motivating than doing so alone. Indeed, peer support has shown positive effects on HE and PA behaviours [41]. Finally, students criticised existing health programs as overly theoretical and called for well-designed SBIs that are practical, engaging, and skill focused. 

Although barriers to HE and PA are often more significant in high deprivation settings, this study and prior research [42] demonstrate that such challenges are present even in LDS, where unhealthy canteen options, limited availability of healthy foods, and affordability issues represent structural issues affecting different socioeconomic groups.

Motivation emerged not only as a barrier, but also as a central mechanism underpinning HE and PA behaviours. Mapping the identified COM-B related barriers to the Behaviour Change Wheel suggests several intervention functions. For instance, low motivation for PA and HE due to limited perceived benefits could be addressed through educational strategies to raise awareness about the importance of these behaviours. In parallel, low motivation for PA related to limited enjoyment could be addressed through persuasion or modelling strategies. Opportunity-related barriers such as the built school environment could be addressed through environmental restructuring to create more supportive environments for HE and PA behaviours. Capability-related barriers, such as lack of trained staff may be addressed through training stretegies [25]. This theoretically informed approach enables mapping of study findings to actionable intervention strategies tailored to the needs of schools in KSA. 

A key strength of this study is its novelty as the first qualitative study to investigate barriers and enablers to HE, PA, and SBIs implementation among female high school students and staff in Riyadh, guided by behaviour change theories. By integrating the COM-B and the SEM, this dual-framework approach enables a theoretically informed interpretation of findings, which extends beyond existing qualitative research in KSA. The inclusion of schools from different deprivation levels captured diverse perspectives. Guided by the COM-B model, the SEM and using framework analysis, the study offers a structured and behaviourally informed perspective on future targets for SBIs in this setting and context, beyond the level of the individual. The qualitative approach captured lived experiences and insights not accessible through quantitative methods.

Limitations include the small sample size and number of schools included, which limits generalisability to other populations such as male students, private schools, or schools in different regions. In addition, the FGDs may have introduced peer influence among students, which could have shaped their responses, representing a potential limitation [28]. Furthermore, while the moderator’s cultural positionality facilitated access to schools and rapport-building with participants, some FGDs perspectives regarding cultural norms around gender, body image, and physical activity may have been influenced by power dynamics between the adult researcher and student participants. Despite this, findings provide valuable insights for schools in KSA and the wider Gulf region with similar socio-cultural contexts.

The present study has several recommendations for the design and implementation of obesity prevention SBIs in KSA in relation to the identified barriers and enablers. Motivation should be considered as a central component driving behaviour change shaped by several factors such as perceived benefits and enjoyment, rather than just a barrier. SBIs should therefore employ behaviour change theories to guide the underpinnings of intervention design, such as the COM-B model, to enhance motivation-related factors (such as peer support, modelling and persuasion), as well as addressing capability-related factors (such as training and education), alongside opportunity-related factors (such as improving HE and PA environments). Effective SBIs implementation requires inter-sectoral collaboration between educational and health sectors, aligning with national policies to improve HE and PA behaviours among adolescents.

Future research should consider the inclusion of male students, other school staff such as canteen providers and Ministry of Education stakeholders, and making a comparison between private and public schools across different regions. Further investigation of cultural barriers to PA among Saudi adolescents is also recommended. These future directions will support the development of tailored, multi-level obesity prevention SBIs in Saudi schools.

## Conclusion

School staff and students in female-only KSA high schools across the socio-economic spectrum reported numerous barriers and enablers across COM-B constructs, highlighting the need for comprehensive obesity prevention SBIs to address the lack of PA and HE provisions in female public schools. However, female high schools face significant challenges regarding a school environment that supports the implementation of obesity prevention SBIs that promote HE and PA. In order to implement obesity prevention SBIs aimed at improving HE and PA behaviours in high schools, policy changes should address organisational challenges that Saudi schools are faced with, support school staff with adequate training, consider the socio-cultural context of KSA, and advocate for policies to improve the school environment and curriculum. Understanding the school context will help support the development of future obesity prevention SBIs.

## Supplementary Information

Supplementary Material 1.

Supplementary Material 2.

Supplementary Material 3.

## Data Availability

The datasets used and/or analysed during the current study are available from the corresponding author on reasonable request.

## Abbreviations

FGD: Focus group discussion
FGDs: Focus group discussions
HE: Healthy eating
HDS: High deprivation schools
KSA: Kingdom of Saudi Arabia
LDS: Low deprivation schools
MDS: Medium deprivation schools
PA: Physical activity
PE: Physical education
PPIE: Personal and public involvement and engagement
PIS: Participant information sheet
SBIs: Obesity prevention school-based interventions
SEM: Social Ecological Model
WHO: World Health Organisation
