# Supplementary material for: Barriers and enablers to obesity prevention in female-only high schools in Riyadh: a qualitative study exploring healthy eating, physical activity and school-based interventions using the COM-B model
Source: BMC Public Health. 2026 Feb 20;26:1020. doi: 10.1186/s12889-026-26568-1 (PMC13032535; doi:10.1186/s12889-026-26568-1)
Supplement: Supplementary file 1 — Supplementary Material 1. [file 12889_2026_26568_MOESM1_ESM.pdf]

Supplementary material (i)

TABLE.1: School staff FGDs topic guide informed by the COM-B model

Type of FGD	COM-B Constructs		
	**Capability**	**Opportunity**	**Motivation**
**With school staff (teachers/principals) to explore barriers of and enablers to implementing obesity prevention school-based interventions**	1- How would you rate the school's capability and preparedness to implement obesity interventions?<br><br>2- How would you rate your capability of implementing an obesity school-based intervention?	1-What are the barriers of implementing school-based obesity interventions?<br><br>2-What are the facilitators of implementing school-based obesity interventions?<br><br>3-Can you describe whether the school environment supports making healthy lifestyle choices?<br><br>4-What are the opportunities given to you by the Ministry of Education to encourage students to adopt healthy lifestyle practices?	1-What motivates you to accept the implementation of a school-based obesity intervention?<br><br>2-What motivates you to encourage students to adopt healthy lifestyles?

TABLE.2: Students' FGDs topic guide informed by the COM-B model

Type of FGD	COM-B Constructs		
	Capability	Opportunity	Motivation
**With high school students to explore barriers and enablers to healthy eating and physical activity**	1- How easy or difficult is it to perform physical activity and why?<br><br>2- How easy or difficult is it to eat healthy foods and why?<br><br>3- When it comes to being successful in engaging in healthy behaviours like eating healthy nutritious food and being physically active, how do you define success?	1- What are the social barriers to maintaining a healthy diet?<br><br>2- What are the physical barriers to maintaining a healthy diet?<br><br>3- What are the social barriers to being physically active?<br><br>4- What are the physical barriers to being physically active?<br><br>5- Can you describe whether your school/home/wider environment supports making healthy lifestyle choices?	1- What motivates you to eat healthy and be physically active?<br><br>2- Can you give us some advice on how to promote healthy dietary behaviours among students?<br><br>3- How can a school-based intervention motivate you to adopt a healthy lifestyle?
