# Supplementary material for: Barriers and enablers to obesity prevention in female-only high schools in Riyadh: a qualitative study exploring healthy eating, physical activity and school-based interventions using the COM-B model
Source: BMC Public Health. 2026 Feb 20;26:1020. doi: 10.1186/s12889-026-26568-1 (PMC13032535; doi:10.1186/s12889-026-26568-1)
Supplement: Supplementary file 2 — Supplementary Material 2. [file 12889_2026_26568_MOESM2_ESM.pdf]

Supplementary material (ii)

COREQ (Consolidated Criteria for Reporting Qualitative Research) Checklist

Domain 1: Research Team and Reflexivity

1. Interviewer/facilitator

Which author(s) conducted the interview or focus group?

[Response: Sarah Aldukair]

2. Credentials

What were the researcher's credentials (e.g., PhD, MD)?

[Response: Master of public health]

3. Occupation

What was their occupation at the time of the study?

[Response: PhD student]

4. Gender

Was the researcher male or female?

[Response: female]

5. Experience and training

What experience or training did the researcher have?

[Response: Dr. Laura McGowan has training in psychology, health psychology, obesity and health behaviour change; Prof. Jayne Woodside in nutrition and public health; Prof. Khalid Almutairi in statistics and public health, and Sarah Aldukair in public health and health education]

6. Relationship established

Was a relationship established prior to study commencement?

[Response: No]

7. Participant knowledge of the interviewer

What did participants know about the researcher?

[Response: A PhD student conducting a qualitative study]

8. Interviewer characteristics

What characteristics were reported about the interviewer/facilitator (bias, assumptions)?

[Response: A female researcher conducting research in a female only school as per cultural norms]

Domain 2: Study Design

9. Methodological orientation and theory

What methodological orientation was stated to underpin the study?

[Response: Qualitative study guided by the COM-B model with framework analysis]

10. Sampling

How were participants selected (e.g, purposive, convenience, snowball)?

[Response: Purposive sampling from high, middle, and low deprivation schools. Eligible students were provided with a participant information sheet (PIS) and parental consent forms; participants were randomly selected alphabetically by surname. Staff received a PIS and were invited to participate based on availability and consent]

11. Method of approach

How were participants approached (e.g., face-to-face, email)?

[Response: E-mails were sent to schools, the school principal was approached through a telephone call to approve access to school, letters were sent to participants (consent forms to teachers and parental consent forms to students)]

12. Sample size

How many participants were in the study?

[Response: 37 students, 19 school staff members]

13. Non-participation

How many people refused or dropped out? Reasons?

[Response: None]

14. Setting of data collection

Where was the data collected?

[Response: Female-only schools in Riyadh]

15. Presence of non-participants

Was anyone else present besides participants and researchers?

[Response: No]

16. Description of sample

What are the important characteristics of the sample?

[Response: Students were aged 16-17]

17. Interview guide

Were questions, prompts, guides provided by authors? Was it pilot tested?

[Response: Questions were developed by authors by creating topic guides. Feedback on the students' topic guide was obtained through an informal personal and public involvement and engagement session with students from outside the studied schools]

18. Repeat interviews

Were repeat interviews carried out?

[Response: No]

19. Audio/visual recording

Did the research use audio or visual recording to collect data?

[Response: Yes]

20. Field notes

Were field notes made during and/or after the interview?

[Response: During]

21. Duration

What was the duration of each interview/focus group?

[Response: Approximately 1 hour]

22. Data saturation

Was data saturation discussed?

[Response: Data saturation was considered; however, it was not used as a primary determinant to the sample size. The number of focus group discussions (n=9) was predetermined to ensure adequate representation of both participants' groups. During framework analysis, consistent codes were observed across the data, suggesting that sufficient information was captured to address the research aims]

23. Transcripts returned

Were transcripts returned to participants for comment or correction?

[Response: No]

Domain 3: Analysis and Findings

24. Number of data coders

How many data coders analyzed the data?

[Response: 3]

25. Description of the coding tree

Did authors provide a description of the coding tree?

[Response: No]

26. Derivation of themes

Were themes identified in advance or derived from the data?

[Response: Codes were derived from the data]

27. Software

What software, if applicable, was used to manage the data?

[Response: NVivo12]

28. Participant checking

Did participants provide feedback on the findings?

[Response: No]

29. Quotations presented

Were participant quotations presented to illustrate themes?

[Response: Yes]

30. Data and findings consistent

Was there consistency between data presented and findings?

[Response: Yes]

31. Clarity of major themes

Were major themes clearly presented in the findings?

[Response: Yes]

32. Clarity of minor themes

Is there a description of diverse cases or minor themes?

[Response: Yes, non-consistent codes]
