# Supplementary material for: Barriers and enablers to obesity prevention in female-only high schools in Riyadh: a qualitative study exploring healthy eating, physical activity and school-based interventions using the COM-B model
Source: BMC Public Health. 2026 Feb 20;26:1020. doi: 10.1186/s12889-026-26568-1 (PMC13032535; doi:10.1186/s12889-026-26568-1)
Supplement: Supplementary file 3 — Supplementary Material 3. [file 12889_2026_26568_MOESM3_ESM.pdf]

Supplementary material (iii)

TABLE.3: Analytic process

Raw quote	Code	COM-B category
‘’It is very difficult to eat healthy because I have to cut the sugars and refrain from foods I like.<br><br>Healthy food is not tasty so it will be hard for me to eat it.’’ (Student 1-MDS)	Difficulty of HE	Capability
‘’Healthy food is expensive and unhealthy food is available and cheap... Healthy alternatives are always expensive.’’<br>(Student 5-LDS)	Affordability	Opportunity
‘’ I just don't have the motivation to prevent myself from eating unhealthy food.’’<br>(Student 1- HDS)	Lack of motivation for HE and PA	Motivation
